# Breast cancer care amidst a pandemic: a scoping review to understand the impact of coronavirus disease 2019 on health services and health outcomes

**DOI:** 10.1093/intqhc/mzad048

**Published:** 2023-07-03

**Authors:** Charlotte Myers, Kathleen Bennett, Caitriona Cahir

**Affiliations:** School of Population Health, RCSI University of Medicine and Health Science, Dublin D02 DH60, Ireland; Data Science Centre, School of Population Health, RCSI University of Medicine and Health Science, Dublin D02 DH60, Ireland; Data Science Centre, School of Population Health, RCSI University of Medicine and Health Science, Dublin D02 DH60, Ireland

**Keywords:** COVID-19, breast cancer, social determinants of health, quality of life, health services, psychosocial

## Abstract

Since the onset of the coronavirus disease 2019 (COVID-19) pandemic, health services for breast cancer (BC) have been disrupted. Our scoping review examines the impact of the COVID-19 pandemic on BC services, health outcomes, and well-being for women. Additionally, this review identifies social inequalities specific to BC during the pandemic. Using the Preferred Reporting Items for Systematic Reviews and Meta-Analyses extension for scoping reviews guidelines, the literature search was conducted using scientific databases starting from March 2020 through November 2021. Studies were identified and selected by two researchers based on inclusion criteria, and the relevant data were extracted and charted to summarize the findings. Ninety-three articles were included in this review. Main themes included are as follows: (i) the impact of COVID-19 on BC services; (ii) the impact of COVID-19 on health outcomes and well-being in women with BC; and (iii) any variation in the impact of COVID-19 on BC by social determinants of health. There were apparent disruptions to BC services across the cancer continuum, especially screening services. Clinical repercussions were a result of such disruptions, and women with BC experienced worsened quality of life and psychosocial well-being. Finally, there were social inequalities dependent on social determinants of health such as age, race, insurance status, and region. Due to the disruption of BC services during the COVID-19 pandemic, women were impacted on their health and overall well-being. The variation in impact demonstrates how health inequities have been exacerbated during the pandemic. This comprehensive review will inform timely health-care changes to minimize long-term impacts of the pandemic and improve evidence-based multidisciplinary needs.

## Introduction

The coronavirus disease 2019 (COVID-19) pandemic has impacted millions of individuals around the world [1]. Health services for cancer have been significantly impacted since the emergence of the COVID-19 pandemic, and the majority of cancer centres worldwide have struggled to provide adequate cancer services, including screening and diagnostic services, active treatment, post-treatment care, and outpatient services, due to precautionary measures against COVID-19 and an overwhelmed health system [2–4].

Breast cancer (BC) is one of the most common cancers, and women with a diagnosis of BC were classified as vulnerable during the pandemic because of their compromised health status [5]. COVID-19 has also negatively impacted women with BC both physically and psychosocially [6, 7]. To inform BC care planning, it would be valuable to better understand how women with BC have being impacted by COVID-19, not only in terms of BC services but also in terms of their well-being, including quality of life (QoL) and their psychosocial health. Such an understanding would inform evidence-based psychosocial care and enable the development of targeted interventions or support services to reduce long-term psychological and physical morbidity from COVID-19 in women with BC.

Evidence is also emerging of potential health inequalities pertaining to COVID-19 [8]. The social determinants of health (SDH) framework details interactions between social and economic factors that may contribute to a greater disruption of health services for certain individuals and negative health outcomes [8, 9]. Prior to the pandemic, research on health inequalities for BC identified the following SDH: income, health insurance, education, region, race, and social support [10, 11]. Understanding the impact of COVID-19 on BC services and women’s well-being within the context of the SDH framework may help identify any patterns of health inequalities pertaining to the pandemic. Therefore, the aim of this scoping review is to examine the impact of COVID-19 on BC services and the health and well-being for women with a diagnosis of BC and how it may vary by SDH.

## Methods

This review followed Preferred Reporting Items for Systematic Reviews and Meta-Analyses (PRISMA) extension for scoping reviews checklist, which defines the necessary items for constructing a scoping review [12] and adhered to the framework methodology of Arksey and O’Malley [13], including the following stages: (i) identifying the research question; (ii) identifying relevant studies; (iii) study selection; (iv) charting the data; and (v) collating, summarizing, and reporting the results.

### Identifying the research question

This scoping review was developed to describe the nature, number, and scope of published research articles measuring the impact of COVID-19 on BC services and the health and well-being for women with a diagnosis of BC and how it may vary by SDH.

The following specific research questions were identified to address the aim of the review:

- How has the COVID-19 pandemic impacted BC services?
- How has the COVID-19 pandemic impacted the health and well-being for women living with and beyond BC?
- Does the impact of the COVID-19 pandemic on BC services and the health and well-being of women with BC vary according to SDH?

### Identifying relevant studies

The literature search was conducted using the following six databases: Embase, PsycINFO, PubMed, CINAHL, Cochrane Library, and Web of Science. The search included only peer-reviewed articles published in the English language after 2020. The electronic search strategy included Medical Subject Headings (MeSH), keywords, and their derivatives (pandemic or COVID-19 or coronavirus) and (breast cancer). The search strategy was conducted by a medical librarian in November 2021 (Appendix 1). All articles were downloaded into EndNote, and the duplicates were removed.

### Study selection

One reviewer (C.M.) screened all identified articles by titles first. Abstracts were then assessed for eligibility by two researchers (C.M. and C.C.) independently. Detailed inclusion and exclusion criteria are found in Table 1. Eligible abstracts were then sought for full-text retrieval, and full-text articles were reviewed independently (C.M. and C.C.). The review team discussed and resolved differences. Furthermore, alerts were created within each database which will allow for future inclusion of relevant articles.

**Table 1.** Inclusion and exclusion criteria.

Study characteristics	Inclusion criteria	Exclusion criteria
Population/participants	Female aged 18+ years women with a diagnosis of BC Women receiving screening services before a diagnosis of BC	Male only Under 18 Studies specific to breast reconstruction surgery only Reporting BC results with other cancer diagnoses without distinct BC results
Study design	Observational studies (retrospective or prospective cohort studies, cross-sectional studies, and case–control studies), qualitative studies, and mixed-methods studies	Literature reviews, clinical trials, genetic testing, and modelling/predictive research studies
Predictor measures	Impact of COVID-19	Non-COVID-19-related
Outcome measures	BC diagnoses/screening services Impacted BC services including active treatment (e.g. surgery, chemotherapy, and radiotherapy) and follow-up care (e.g. routine care) Clinical outcomes (e.g. treatment prognosis) QoL Psychosocial well-being (e.g. depression, anxiety, and fear of cancer recurrence)	COVID-19 infection Change in treatment due to COVID-19 infection COVID-19 outcomes

### Charting the data

Data extraction for each full-text article followed corresponding guidelines specific to the study design [14–16]. After both researchers (C.M. and C.C.) independently reviewed the selected full-text articles, data were initially extracted from the first five studies and compared by the two reviewers to ensure consistency. The following data were extracted and charted: study design, country, participant characteristics, recruitment method/sample size, time period/phase of lockdown, and main results. Main results were charted to align with the impact of COVID-19 on BC services and/or the impact of COVID-19 on health and well-being separately; for studies that examined and reported on the impact of COVID-19 in relation to SDH, additional data were extracted (e.g. age, race/ethnicity, insurance status, and region).

### Collating, summarizing, and reporting

**Figure 1:**
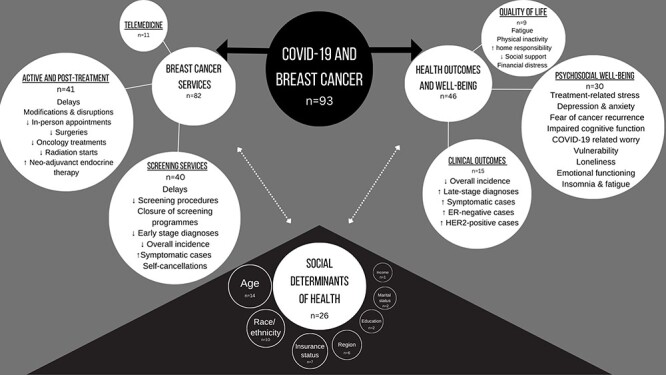
The relationship between COVID-19 and SDH on breast cancer services and health outcomes and well-being.

The data from the included studies were collated to provide both descriptive and numerical summary of the findings. To comprehensively understand the impact of COVID-19 on BC, the findings were coded to the three pre-established themes, which correspond with each research question, respectively. A number of sub-themes were then established for each of the three themes: (i) impact of COVID-19 on BC services (screening and diagnostic services, treatment and follow-up care, and telemedicine); (ii) impact of COVID-19 on health outcomes and well-being in women with BC (clinical outcomes, QoL, and psychosocial well-being); (iii) variation in the impact of COVID-19 on BC services and health and well-being by SDH (age, race/ethnicity, insurance status, region, education, marital status, and income). Results are presented narratively by themes and sub-themes in Tables 2–4, and Fig. 1 illustrates a visual summary presentation of the results.

**Table 2.** The impact of COVID-19 on BC services (Research question 1).

Primary author, year	Study design	Country	Participant characteristics	Recruitment method/sample size	Time period, phase of lockdown	Results	Sub-themes
BC services (screening, active/post-active treatment, and telemedicine)							
Amornsiripanitch, 2021 [17]	Longitudinal (retrospective)	The USA	All BC patients within a scheduling database (i) Female (ii) Age range 24–101 years (mean age: 60.8)	*n* = 15 792 (pre-COVID) *n* = 16 595 (COVID)	June–August 2019/ March–June 2020/ June–August 2020	The cancellation of screening appointments was higher during COVID reopening times (46%) compared to pre-COVID times (37%)	Screening and diagnostic services
Amram, 2021 [18]	Longitudinal (retrospective)	The USA	Women who completed BC screening (i) Female only (ii) Mean age: 62.4 ± 11.1 years	*n* = 55 678 (pre-COVID) *n* = 27 522 (COVID)	April–December 2019/2020	Completed screening appointments drastically dropped (49%) from pre-COVID (2019) to post-COVID (2020)	Screening and diagnostic services
Bessa, 2021 [19]	Longitudinal (retrospective)	Brazil	Women who received mammograms (i) Female only (ii) Age range 50–69 years	*n* = 1 939 415 (pre-COVID) *n* = 1 126 688 (COVID)	2019/2020	Mammograms decreased by 42% from 2019 to 2020, yet mammograms for women with palpable lumps slightly increased from 2019 to 2020	Screening and diagnostic services
Bonadio, 2021 [20]	Longitudinal (retrospective)	Brazil	Patients with a diagnosis of BC	*n* = 457 (pre-COVID) *n* = 268 (COVID)	September–January 2019–20/2020–21	The proportion of diagnoses made from routine screening services decreased during the pandemic (25.5–13.7%), yet the interval time from diagnosis to treatment decreased during the pandemic	Screening and diagnostic services
Chou, 2021 [21]	Longitudinal (retrospective)	Taiwan	Patients receiving biopsies from positive screening (i) Female only (ii) Mean age: 51.1 ± 11.7 years (pre-COVID) and 51.2 ± 12.8 years (COVID)	*n* = 430 (pre-COVID) *n* = 355 (COVID)	January–July 2019/2020	The number of breast biopsies decreased during COVID-19, and the total number of diagnosed BC cases decreased during the pandemic, especially those of early stages	Screening and diagnostic services
Chou, 2021 [22]	Longitudinal (retrospective)	Taiwan	Women receiving mammograms Mean age: 55.1 ± 1.7 years (pre-COVID) 51.2 ± 4.4 years (COVID)	*n* = 4816 (pre-COVID) *n *= 3041 (COVID)	January–May 2019/2020	37% of fewer mammograms were recorded during the COVID-19 pandemic compared to 1 year prior, and the weeks with higher difference of mammograms corresponded with weekly COVID-19 cases. Differences were detected for self-requested mammograms and screening mammograms, but not diagnostic mammograms	Screening and diagnostic services
Collado‐Mesa, 2020 [23]	Longitudinal (retrospective)	The USA	Monthly breast imaging procedures	*n* = 7190 (pre-COVID) *n* = 1090 (COVID)	April 2018/2019/2020	All facilities experienced a decrease in breast procedures during COVID-19, including screening, diagnostic, ultrasound, magnetic resonance imaging (MRI), and biopsies. These decreases were more apparent in facilities with COVID-19 rescheduling measures	Screening and diagnostic services
Crivellaro, 2021 [24]	Longitudinal (retrospective)	Canada	Patients presenting for diagnostic mammography Pre-COVID: age range 30–85 years, median age: 57 years Female *n* = 62, male *n* = 2 COVID: age range 25–80 years, median age: 50 years Female *n* = 74, male *n* = 1, transgender *n* = 2	*n* = 64 (pre-COVID) *n* = 77 (COVID)	April–June 2019/2020	The total number of patients presenting to the breast clinic was significantly lower during the pandemic, yet the percentage of referred non-screening-related mammography was greater during the pandemic. The diagnostic interval was lower in the pandemic group compared to the pre-pandemic group	Screening and diagnostic services
DeGroff, 2021 [25]	Longitudinal (retrospective)	The USA	Women receiving screening tests within national programme providing early detection for women of without health insurance	*n* = 487 645	January–June 2015–20	BC screening services declined during 2020 compared to the 5-year average, with the biggest decline apparent in April 2020 with an 87% decline. Screening services recovered by June 2020	Screening and diagnostic services
Eijkelboom, 2021 [26]	Longitudinal (retrospective)	The Netherlands	Women with a diagnosis of BC —50–74 years of age	*n* = 5306 (2020–COVID) *n* = 7302 (2019–COVID) *n* = 7250 (2018–COVID)	Weeks 2 through 35 of 2020/2019/2018	The number of diagnoses during 2020 was lower than in the same period of 2018/2019. The number of diagnoses recovered with the recommencement of screening programmes in the later months of the pandemic year but not to pre-pandemic levels	Screening and diagnostic services
Fedewa, 2021 [27]	Longitudinal (retrospective)	The USA	Screening rates for women receiving BC screening among community health centres— 50–74 years of age	49.6% (2020–COVID) 53.9% (2019) 45.8% (2018)	July 2020/ July 2019/ June 2018	BC screening rates rose by 18% from 2018 to 2019 and then declined by 9% from 2019 to 2020	Screening and diagnostic services
Fisher-Borne, 2021 [28]	Cross-sectional	The USA	Cancer clinics participating in federal funded screening programmes	*n* = 13 BC clinics	August–September 2020	BC clinics experienced a 77% reduction in screenings during the pandemic	Screening and diagnostic services
Gathani, 2021 [29]	Longitudinal (retrospective)	UK	BC patients within the National Health Service	*n *= 322 994 (pre-COVID) *n *= 231 765 (COVID)	2019/2020	Cancer wait time (being a surrogate for new diagnoses) decreased during the pandemic year by 28%, and the proportion is larger for non-urgent referrals indicating prioritization for urgent BC referrals	Screening and diagnostic services
Kaltofen, 2020 [30]	Longitudinal (retrospective)	Germany	BC patients (i) Median age: 54.7 years (COVID) (ii) Median age: 56.6 years (pre-COVID)	*n* = 327 (2020–COVID) *n* = 365 (2019–pre-COVID)	January–June 2020/2019	BC diagnoses decreased by 12% during the pandemic year	Screening and diagnostic services
Kang, 2021 [31]	Longitudinal (retrospective)	South Korea	Patients receiving BC screening services and surgical treatment (i) Median age: 53 years	*n* = 1369 (2020–COVID) *n* = 1669 (2019–pre-COVID)	February–July 2020/2019	There was a 9.9% reduction in diagnoses during the pandemic year, especially early on in the pandemic	Screening and diagnostic services
Kirkegaard, 2021 [32]	Qualitative	Denmark	Women eligible for BC screening (i) Female only (ii) Mean age: 62 years	Convenience sample, *n* = 33	April 2020	Women who postponed/cancelled their mammography were motivated to be screened, except in times of high COVID-19 infection rates and when government recommendations suggested against it. Many women balanced the risk of COVID-19 versus the risk of BC in deciding whether to attend mammography	Screening and diagnostic services
Kiziltan, 2021 [33]	Longitudinal (retrospective)	Turkey	BC patients (i) Female only (ii) Age range: 20–82 years (median age: 51)	*n* = 250 (pre-COVID) *n* = 146 (COVID)	Pre-pandemic versus March–June 2020	The number of patients with a diagnosis of BC decreased during the pandemic year compared to pre-pandemic year. More women had a diagnosis prior to the pandemic, yet the percentage of women who were diagnosed was greater within the group of women seen during the pandemic and referrals were similar in both groups	Screening and diagnostic services
Knoll, 2021 [34]	Longitudinal (retrospective)	Austria	Newly diagnosed BC and gynaecological cancer patients (i) Median age = 63 years (pre-COVID) (ii) Median age = 60 years (COVID)	*n* = 495 (pre-COVID) *n* = 394 (COVID) Breast only (both time points): *n* = 596	March–December 2019/2020	There was a 52% reduction in new cases for BC, and there was an inverse correlation between the number of COVID-19 cases in the state with newly diagnosed BC case	Screening and diagnostic services
Li, 2021 [35]	Cross-sectional	The USA	Women attending BC screening Majority in the age range of 40–70 years	*n *= 570	December 2020–January 2021	11% of women reported a delay in their BC screening during the period of December 2020–January 2021. Women with a personal history of COVID-19 exposure/infection tended to report more delays in screening appointments	Screening and diagnostic services
Linck, 2021 [36]	Longitudinal (retrospective)	France	BC diagnoses (i) Pre-COVID mean age: 66 years (ii) COVID mean age: 64 years	*n* = 120 (pre-COVID) *n* = 134 (COVID)	2019/2020	BC diagnoses, respectively, decreased by 26% and 20% during the lockdown and increased by 37% and 48% during the post-lockdown period	Screening and diagnostic services
Miller, 2021 [37]	Longitudinal (retrospective)	The USA	Patients receiving screening mammograms (i) Pre-COVID: median age: 62 years (ii) COVID: median age: 62 years	*n* = 10 757 (pre-COVID) *n* = 9062 (COVID)	March–October 2019/2020	The number of screening mammograms decreased from pre-COVID to COVID, and this reduction was greatest during the period in time when screening services were closed	Screening and diagnostic services
Nyante, 2021 [38]	Longitudinal (retrospective)	The USA	Women receiving BC screening services (i) Mean age: 59 ± 2 years	*n* = 39 444	January 2019 to 2 March 2022/3 March 2020 to 30 September 2020	Examinations reduced during the pandemic. Specifically, screening mammography and diagnostic mammography significantly decreased with the onset of the pandemic and biopsies decreased a few months into the pandemic. Women with higher risk were prioritized for screening services during the pandemic	Screening and diagnostic services
O’Brien, 2021 [39]	Longitudinal (retrospective)	Ireland	Women receiving BC screening services	*n* = 2111 (COVID) *n* = 3008 (pre-COVID)	February–July 2020/ February–July 2018/2019	The number of new patients seen at the breast clinic for symptomatic screening reduced by 30% during the pandemic year, corresponding with a reduction in new diagnoses by 50%. A larger impact on new diagnoses was seen earlier on during the pandemic, and compensation was seen later in 2020	Screening and diagnostic services
Peng, 2020 [40]	Longitudinal (retrospective)	Taiwan	Women attending population-based BC screening	n/a	January–May 2017/2018/2019/2020	The number of women with a positive mammogram undergoing screening and the diagnostic referral rate decreased starting March 2020. More women use mobile mammography services compared to hospital settings, but the number of mammograms conducted in both the hospital setting and mobile units dropped at the beginning of the pandemic	Screening and diagnostic services
Schifferdecker, 2021 [41]	Qualitative	USA	Women eligible for mammography, with and without a BC diagnosis (i) Mean age: 57 years	*n* = 30 (*n* = 22 without BC, *n* = 8 with BC)	March–August 2020	Women described screening experiences as safe and communication strategies around scheduling and appointment procedures varied widely	Screening and diagnostic services
Song, 2021 [42]	Longitudinal (retrospective)	The USA	Women receiving mammograms (i) Aged 40 years and older	n/a	2018/2019/2020	Diagnostic and screening mammograms fell during the onset on the pandemic in 2020. Diagnostic mammograms recovered while screening mammograms lagged in recovery. Women who received mammograms during 2020 were older women, had a history of BC, and more time elapsed between previous mammogram	Screening and diagnostic services
Sprague, 2021 [43]	Longitudinal (prospective)	The USA	Screening and diagnostic mammograms (i) Female only	*n* = 461 083 screening mammograms *n* = 112 207 diagnostic mammograms	January 2019–July 2020	Screening and diagnostic mammograms decreased with the onset of the pandemic and recovered several months later for most groups	Screening and diagnostic services
Sprague, 2021 [44]	Cross-sectional	The USA	Breast care facilities	*n* = 77 facilities	March–September 2020	97% of facilities reported closures or operating at a reduced capacity during COVID-19, with a full recovery by September 2020 in 86% of facilities. Screening mammograms, screening ultrasound, and screening MRIs were the most frequent disrupted services. To facilitate safe treatment services, precautions were taken such as COVID-19 testing and personal protective equipment	Screening and diagnostic services
Toyoda, 2021 [45]	Cross-sectional	Japan	Women with a scheduled screening appointment (i) Age range 30–79 years	*n* = 1874	April–May 2020	26% of women postponed or cancelled their screening appointment from March to April 2020	Screening and diagnostic services
Tsai, 2020 [46]	Longitudinal (retrospective)	Taiwan	Number of breast screenings	*n* = 396 371 (pre-COVID) *n* = 308 463 (COVID)	January–April 2019/2020	The total number of screenings dropped 22% from 2019 to 2020. In-hospital examinations were most impacted, and examinations in outreach and mobile units stayed stable. Recall rates increased during 2020, meaning more of women were called back for further examination	Screening and diagnostic services
Velazquez, 2021 [47]	Cross-sectional	The USA	Performed mammograms (i) Female only	*n* = 9291	January 2019–January 2021	The number of screening mammograms decreased from pre-COVID to COVID and the number of missed appointments also increased. This trend aligned with the lockdown phases during 2020, and mobile mammography was discontinued during a portion of the lockdown. Mammograms recovered after lockdown phases	Screening and diagnostic services
Vrdoljak, 2021 [48]	Longitudinal (retrospective)	Croatia	Newly diagnosed BC patients	*n* = 2535 (2017) *n* = 2651 (2018) *n* = 2875 (2019) *n* = 2848 (2020)	2017/2018/ 2019/2020	After the initiation of hospital lockdowns in 2020, the number of newly diagnosed BC cases decreased. Specifically, there was a 24% decreased in diagnosed cases from March to June, but he impact was not as strong throughout the rest of 2020	Screening and diagnostic services
Wilke, 2021 [49]	Cross-sectional	The USA	BC patients (on behalf of breast surgeons) (i) Mean age: 62.7 years	*n* = 2791	March 2020–March 2021	Most surgeons reported stopping mammogram services at some point during the pandemic; all surgeons reported stopping some portion of surgery during the pandemic. The average wait time to surgery was 44.5 days. There was an increase in neoadjuvant endocrine therapy for estrogen receptor positive (ER+) and human epidermal growth factor receptor 2 positive (HER2+) patients, and 12% of patients who underwent surgery will require further surgery	Screening and diagnostic services Treatment and follow-up care
Tonneson, 2021 [50]	Longitudinal (retrospective)	The USA	BC patients with a new diagnosis (i) Pre-COVID 373 females/3 males Mean age: 60.5 years (ii) COVID Female only Mean age: 59.8 years	*n* = 376 (pre-COVID) *n* = 197 (COVID)	March–August 2019/2020	The methods of detection between 2 years did not differ greatly; neither did the type of surgery performed. The use of neoadjuvant endocrine therapy was higher during COVID-19 times, particularly among women with HER2+ diagnosis	Screening and diagnostic services Treatment and follow-up care
Toss, 2021 [51]	Longitudinal (retrospective)	Italy	Patients diagnosed with BC (i) Pre-COVID 221 females/2 males (ii) COVID 174 females/3 males	*n* = 223 (2019) *n* = 177 (2020)	May–July 2019/2020	Due to the closing of screening programmes, more patients were diagnosed through mammographic follow-up and less patients were diagnosed in situ. There were no significant differences in time to treatment	Screening and diagnostic services Treatment and follow-up care
Eijkelboom, 2021 [52]	Longitudinal (retrospective)	The Netherlands	Women with a recent diagnosis of BC Similar age distribution between years	*n* = 5685 (2018–pre-COVID) *n* = 5838 (2019–pre-COVID) *n* = 4769 (2020–COVID)	Weeks 2 through 17 of 2018/2019/2020	The incidence of BC in 2020 decreased after government lockdown. Fewer women underwent surgery, while more women underwent hormonal treatment. Chemotherapy was less likely for women diagnosed during the earlier months of the pandemic	Screening and diagnostic services Treatment and follow-up care
Koca, 2021 [53]	Longitudinal (retrospective)	Turkey	Patients with a BC diagnoses (i) 146 women, 2 men (ii) Age range: 22–91 years; median age: 51.2 years	Overall: *n* = 148 *n* = 70 (pre-COVID) *n *= 78 (COVID)	2019/2020	The amount of patients seen in the breast clinic (including surgeries) decreased during the pandemic, along with a decrease in screen mammography. Neoadjuvant chemotherapy was higher during the pandemic, along with modified mastectomies	Screening and diagnostic services Treatment and follow-up care
Li, 2020 [54]	Longitudinal (retrospective)	China	Women with early BC Median age: 50 years	*n* = 8397	January–March 2020	Diagnoses were lower in regions with high-infection rates (e.g. Hubei). Surgery decreased in specific regions, and the least impacted treatment was postoperative endocrine therapy. Time-to-treatment intervals increased during the pandemic, along with delays. Such delays were more common in high-infection area	Screening and diagnostic services Treatment and follow-up care
Mathelin, 2021 [55]	Cross-sectional	Multiple	BC clinicians	*n* = 45	June–July 2020	69% of participants reported suspension of screening programmes during the pandemic. 22% of participants reported suspension of breast MRI imaging. Surgeries were postponed mostly for reconstruction and benign tumours or for women with multiple comorbidities. Chemotherapy and radiotherapy were adapted for patients as well. Telemedicine was also preferred by BC clinicians	Screening and diagnostic services Treatment and follow-up care Telemedicine
Pairawan, 2021 [56]	Longitudinal (retrospective)	The USA	Women who cancelled their mammograms	*n* = 2784	January–August 2020	Cancellations increased in the height of the pandemic (March) and then levelled to pre-pandemic levels. Appointments dropped during this time as well. Reasons for cancelled appointments varied by month and mostly were attributed to the patient. The use of electronic portal continued to increase throughout 2020, indicating an increase in telemedicine	Screening and diagnostic services Telemedicine
Soriano, 2021 [57]	Cross-sectional	The USA	Patients with a diagnosis of BC in the USA with postponed surgery (i) Female only (ii) Mean age: 60.1 ± 13.2 years	*n* = 50	June–August 2020	One-third of women were still awaiting surgery even after the clinic resumed surgery procedures and there was a poorer indication of satisfaction and communication with their BC care team	Treatment and follow-up care
Cadili, 2021 [58]	Longitudinal (retrospective)	Canada	Women receiving surgical procedures in a breast clinic (i) Mean age: 60 years (pre-COVID) and 57 years (COVID)	*n* = 99 (pre-COVID) *n* = 162 (COVID)	March–April 2019/2020	More women with a diagnosis of BC were seen for breast surgical procedures during the pandemic and the wait times to surgery decreased during the pandemic. Treatment adaptation included regional anaesthesia and same-day discharge	Treatment and follow-up care
Chen, 2021 [59]	Cross-sectional	China	Patients with BC receiving active treatment (i) Majority (65.1%) aged 46 years and above	*n* = 834	March 2020	Over half of the patients reported affected treatment during the pandemic	Treatment and follow-up care
Choobin, 2021 [60]	Cross-sectional	Iran	BC patients with a primary diagnosis of BC in Iran (i) Female only (ii) Mean age: 45.9 ± 9.6 years	Volunteer recruitment, *n* = 139	May 2020	About 30% of women indicated delayed BC services and those women expressed higher emotional vulnerability and worry to COVID-19	Treatment and follow-up care
Dauti Işıklar, 2021 [61]	Longitudinal (retrospective)	Turkey	Newly diagnosed BC patients Median age for 2020: 50 years	*n* = 428 (2017–pre-COVID) *n* = 702 (2018–pre-COVID) *n* = 610 (2019–pre-COVID) *n* = 521 (2020–COVID)	May–June 2020	The number of patients seen in the single clinic declined during the height of the pandemic, regardless of diagnostic stage. Less than half of BC patients received chemotherapy and endocrine therapy was more common during the pandemic than chemotherapy	Treatment and follow-up care
Fedele, 2021 [62]	Cross-sectional	Italy	Oncologists who provided treatment to women with metastatic BC	*n* = 90	May–June 2020	Main changes to cancer treatment included a decrease in monotherapy administration and an increase in oral anti-cancer drugs. These changes were heterogeneous for different cancer types	Treatment and follow-up care
Filipe, 2020 [63]	Longitudinal (retrospective)	The Netherlands	BC patients who underwent breast surgery during the pandemic (i) Mean age 62.2 ± 13.1 years	*n* = 217	March–May 2020	The number of total surgeries decreased throughout government lockdown in 2020	Treatment and follow-up care
Fortunato, 2021 [64]	Cross-sectional	Italy	Breast care units treating women newly diagnosed with BC	*n* = 100 breast care units, treating 37 678 BC patients	April 2020	There was a major decrease in BC services, including radiology, surgery, oncology, and radiotherapy. There was a decrease in available hospital space for BC services, and many breast units indicated modifications to the organization of their unit	Treatment and follow-up care
Gasparri, 2020 [65]	Cross-sectional	41 European Union countries	Breast centres	*n* = 377	April 2020	One-third of breast units reported a decrease in overall BC workload by at least 50%, and there was a significant increase in time between diagnosis and treatment. Additionally, the majority of breast units indicated modified chemotherapy protocols, and the majority of breast units preferred endocrine therapy to surgery	Treatment and follow-up care
Hawrot, 2021 [66]	Longitudinal (retrospective)	The USA	Newly diagnosed, early-stage BC patients (i) Majority 50–70 years of age	*n* = 164 (2020–COVID) *n* = 202 (2018–COVID)	January–May 2020/2018	The amount of patients seen at the participating breast clinics decreased during the pandemic year. Diagnoses were more likely to be of late stage, and preoperative systemic therapy was more common	Treatment and follow-up care
Koch, 2020 [67]	Longitudinal (retrospective)	Canada	Number of radiation therapy starts	*n *= 160 (pre-COVID) *n* = 118 (COVID)	March–April 2019/2020	The amount of breast radiation starts decreased 39% from 2019 to 2020. Adaptation of radiation therapy included regional nodal irradiation, and there was an increase of such treatment type	Treatment and follow-up care
Lee, 2020 [68]	Longitudinal (retrospective)	South Korea	Newly-diagnosed BC patients scheduled for surgery (i) 61 females, 1 male (ii) Mean age: 55.7 ± 11.2 years	*n* = 62 years	February–April 2020	Patients in the delayed group experienced an average delay to surgery of almost 16 days. Patients who did not experience had planned mastectomy and/or received needle biopsy for diagnosis. Those who did experience delays with planned small-scale surgery and good prognosis of BC	Treatment and follow-up care
Lee, 2022 [69]	Longitudinal (retrospective)	South Korea	BC patients receiving radiation therapy (i) pre-COVID median age: 55 years (ii) COVID median age: 49 years	*n* = 287 (pre-COVID) *n* = 279 (COVID)	January–July 2019/2020	Interruption of radiation did not change much during the pandemic year compared to 2019, but reasons for delays varied between 2 years. Patients indicated delays caused by COVID-19 screening and/or clinic-related interruptions which were typically a longer delay than other causes	Treatment and follow-up care
Murris, 2021 [70]	Cross-sectional	France	BC centres	*n* = 12	March–May 2020	50% of BC centres increased surgical procedures during the beginning months of the pandemic. Postponements were more common than cancellations for varying BC interventions. Surgery was shifted to an outpatient setting and hospitalization duration reduced during the pandemic	Treatment and follow-up care
Rocco, 2021 [71]	Mixed-methods	Multiple	BC surgeons	*n* = 12 (interview) *n* = 100 (survey)	April 2020	Some countries identified specific guidelines to BC surgery during the pandemic, while others did not. BC screening was halted in most countries, and most had a triage system. Separation from COVID wards was used by most clinics and countries required COVID testing before procedures. Most countries shifted to emergency surgeries only and in-person appointments dropped	Treatment and follow-up care
Romics, 2021 [72]	Longitudinal (prospective)	Scotland	BC patients undergoing surgery (i) Median age: 54 years	*n* = 179	March–May 2020	Breast conservation rate was higher among pre-COVID surgeries; there was no immediate reconstruction surgery during COVID. The length of hospital stay was shorter during COVID, and there was a higher proportion of patients who received neoadjuvant chemotherapy during COVID-19	Treatment and follow-up care
Savard, 2021 [6]	Qualitative	Canada	Women with non-metastatic BC who were scheduled to have chemotherapy (i) Female only (ii) Mean age: 51.8 years	Volunteer sample, *n* = 23	May 2020	Women expressed changes in cancer care, including delays and cancellations	Treatment and follow-up care
Swainston, 2020 [73]	Cross-sectional	The UK	Patients diagnosed with primary BC (i) Female only (iii) Mean age: 51 years	Volunteer sampling via support platforms, *n* = 234	2020	32% had been impacted by disruption to their scheduled oncology services	Treatment and follow-up care
Syed, 2020 [74]	Case–control	India	BC patients on neoadjuvant therapy awaiting surgery (i) Mean age: 45.35 ± 10.6 years	*n* = 54	March–August 2020	The time interval from the last chemotherapy treatment to surgery increased among patients. Patients experienced a duration delay of about 89 days; therefore, many needed re-imaging to investigate the current status of disease	Treatment and follow-up care
Vanni, 2020 [75]	Longitudinal (retrospective)	Italy	BC patients awaiting surgery (i) Pre-COVID median age: 62 years (ii) COVID median age: 60.8 years	*n* = 209 (pre-COVID) *n* = 223 (COVID)	March–May 2019/2020	The waiting time from examination to surgery significantly increased from 2019 to 2020. Waiting time was a predictive measure for lymph node involvement	Treatment and follow-up care
Vanni, 2020 [76]	Longitudinal (retrospective)	Italy	Patients attending breast centre Suspicious breast lesion (i) Pre-COVID mean age: 57.6 (ii) COVID mean age: 59.5 years BC diagnosis (i) Pre-COVID mean age: 64.3 years (ii) COVID mean age: 61.2 years	*n* = 82 (suspicious breast lesion) *n* = 78 (BC group)	January–March 2020	Women from both clinical groups were more likely to refuse BC procedures post-COVID-19 due to risk of COVID-19 infection	Treatment and follow-up care
Villarreal-Garza, 2021 [77]	Cross-sectional	Mexico	Women with a diagnosis of BC	*n* = 142	May–August 2020	83% of women indicated disruption to their BC care, and only 39% of those women indicated recommencement of treatment. Median time for treatment to recommence was 60 days	Treatment and follow-up care
Satish, 2021 [78]	Cross-sectional	The USA	BC patients (i) 348 females/2 males (ii) Mean age: 57.1 ± 14 years	*n* = 350	February–April 2020	65% of patients experienced some sort of delay or change in their cancer treatment. 43% of delays were attributed to COVID-19 and 37% were non-COVID-related. The median COVID-19-related delay in systemic therapy was 24.5 days. Practice delays and practice modification were the most frequent COVID-related delays. Delays were more frequent among early-stage diagnoses	Treatment and follow-up care
Shinan-Altman, 2020 [79]	Cross-sectional	Israel	BC patients (i) Female only (ii) Mean age: 51 years	*n* = 151	April 2020	30% of women cancelled an appointment during COVID-19	Treatment and follow-up care
Hamlish, 2021 [80]	Cross-sectional	The USA	BC survivors (i) Mean age: 47.9 ± 10.9 (ii) Race: 81% White; 19% Black	Snowball sampling via social media and email, *n = *570	April 2020	42% of respondents were experiencing some sort of delay for their BC treatment	Treatment and follow-up care
Alipour, 2020 [81]	Cross-sectional	Iran	Patients who were scheduled to have a BC appointment but did not attend their visit (i) Female (ii) Age range 19–64 years (mean age: 42.9)	Random selection of 10% of patient list, *n* = 134	March–May 2020	The average appointment delay was about 3 months. Reasons for delays included fear of COVID-19 transmission, being unable to make the appointment (e.g. government restrictions) and personal reasons	Treatment and follow-up care
Zhang, 2021 [82]	Qualitative	The USA	Women participating in online forums within Breastcancer.org, a large BC support community in the USA	Unrestricted, volunteer sampling, *n* = 192	March–April 2020	Topics discussed included self-cancellation, diagnosis and treatment delays, new surgical experiences, and health-care changes	Treatment and follow-up care
Zhao, 2021 [83]	Cross-sectional	The USA	BC patients (i) Mean age (White): 61.6 ± 11.6 years (ii) Mean age (Black): 63.6 ± 11.9 years	*n* = 1300	July–September 2020	25% of the population struggled to receive treatment during the pandemic	Treatment and follow-up care
Kim, 2021 [84]	Cross-sectional	South Korea	Patients diagnosed with BC within the last 2 years (i) Age: 42.8 ± 7.7 years	*n* = 154	April–June 2020	18.8% of women reported changes to their BC treatment, most of which being delays	Treatment and follow-up care
Kennard, 2021 [85]	Longitudinal (prospective)	The USA	BC patients receiving active treatment (i) Mean age: 60.6 ± 13.3 years	*n* = 73	March–June 2020	56% of patients had no change in treatment, while 44% did experience change in recommended treatment. All women were informed of their change in treatment, and most women in both groups experienced some sort of telehealth service. The delay from diagnosis to surgery was much longer for women who experienced a change of treatment	Treatment and follow-up care Telemedicine
Mo, 2021 [86]	Longitudinal (retrospective)	The SA	Early-stage BC survivors 2812 females/18 males	*n* = 2830	2019–2020	Over half of the patients who attended a follow-up visit in 2019 did not attend an in-person or telehealth appointment during 2020. Patient who attended follow-up visits were more likely to have received radiotherapy in the past	Treatment and follow-up care
Papautsky, 2020 [5]	Cross-sectional	The USA	BC survivors (i) 608 females/1 male (ii) Mean age: 47.8 years	Snowball sampling via social media, *n* = 609	April 2020	44% of women indicated some sort of delay to their regular cancer care during the pandemic. BC survivors reported delays in appointments such as follow-up visits, reconstruction, and diagnostic imaging	Treatment and follow-up care
Seven, 2021 [87]	Qualitative	Turkey	BC survivors who completed treatment within the last 5 years (i) Female only (ii) Mean age: 51 ± 5.9 years	Convenience sample, *n* = 18	May 2020	Women experienced changes to their routine/follow-up care	Treatment and follow-up care
Helm, 2020 [7]	Cross-sectional	USA	BC survivors (i) Female only (ii) Mean age: 55.3 ± 9.8 years	*n* = 15	May 2020	Closing of rehabilitation services reduced QoL for BC survivors, but 33% received telehealth services	Treatment and follow-up care Telemedicine
Bizot, 2021 [88]	Cross-sectional	France and Italy	Patients with BC (i) Majority (72.6%) aged ≥50 years	*n* = 1299	April–May 2020	Patients with telehealth services did not experience much anxiety and patients reported satisfaction with telehealth services. This satisfaction was correlated with age (lower satisfaction in older groups), modality of service (women expressed higher satisfaction for video consultations compared to telephone), and anxiety score (low satisfaction for high anxiety)	Telemedicine
Bruce, 2021 [89]	Mixed-methods	The UK	BC patients with a scheduled telephone consultation, who had undergone breast surgery (i) Mean age: 61.5 ± 12.0 years	Random selection, *n* = 30	n/a	Most patients were satisfied with their telephone consultations, although half of the patients would have preferred face-to-face consultations	Telemedicine
Johnson, 2021 [90]	Cross-sectional	The USA	BC patients in an ambulatory setting (i) Age range 25–83 years; median age: 63 years	*n* = 75	June–September 2020	On a 1–7 Likert scale, patients scored 5.5 overall for satisfaction of telehealth services during the pandemic. This was slightly lower (4.75) for surgical oncology visits. Patients scored similarly for usability of telehealth services. There was also a strong correlation between satisfaction and usability	Telemedicine
Ludwigson, 2022 [91]	Mixed-methods	The USA	BC patients (i) Majority over the age of 45	*n* = 133 (survey) *n* = 20 (interview)	August 2020–February 2021	63% of women attended a telehealth service for their BC care, and the majority were satisfied with their appointment	Telemedicine
Picardo, 2021 [92]	Cross-sectional	Italy	BC and gynaecological cancer patients receiving follow-up visits via telemedicine (i) Female only (ii) Median age of BC group: 67 years	*n* = 345 (*n* = 267 BC, *n* = 78 gynaecological cancer)	March–May 2020	Telemedicine was accepted well among women with BC during early lockdown phases	Telemedicine
Sonagli, 2021 [93]	Longitudinal (retrospective)	Brazil	Women with scheduled telemedicine appointment (i) Female only (ii) Mean age: 52.8 years	*n* = 77	June–October 2020	Women had telemedicine visits for the following purposes: follow-up, screening for benign disease, second opinion, and general guidance. Of these women, over half were referred through for further follow-up/surveillance	Telemedicine
Zimmerman, 2020 [94]	Cross-sectional	The USA	Breast and gynaecologic centre patients	*n* = 215	March–June 2020	35% of patients reported use of telemedicine and of those patients, 92% reported satisfaction. Patient reported satisfaction with telemedicine in the following ways: saves time, increases access to care, and improves health	Telemedicine

## Results

### Study population

**Figure 2:**
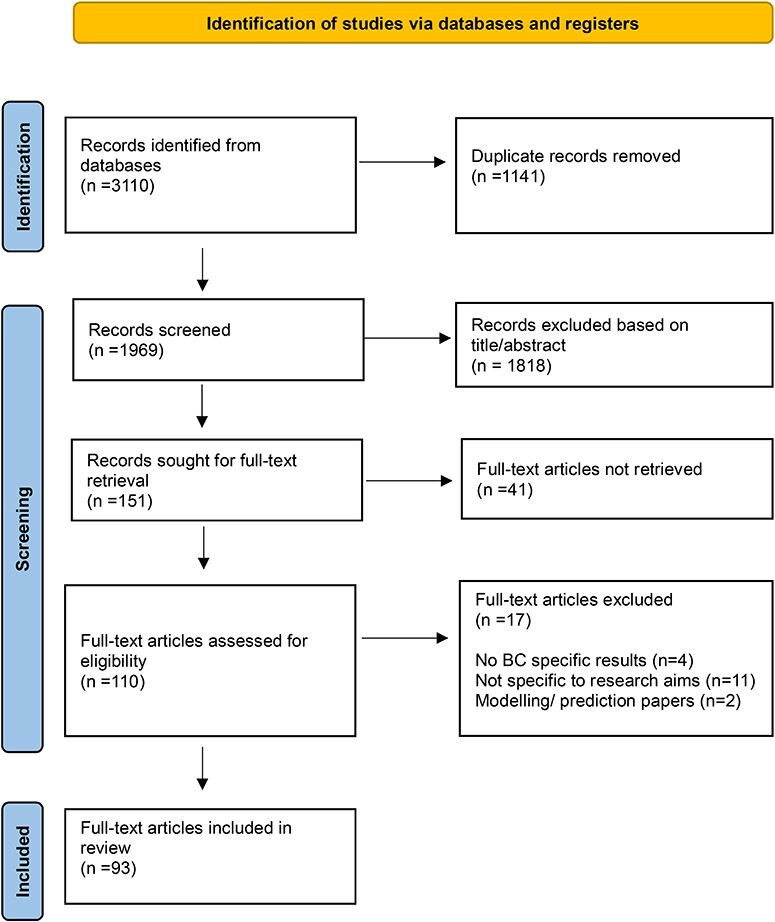
PRISMA flow diagram for study selection and inclusion.

A total of 3110 records were identified and 1969 titles were screened. Of these, 465 abstracts were assessed for eligibility and 151 articles were then sought for full-text retrieval; 93 articles were eligible for inclusion. Figure 2 displays the PRISMA extension for scoping reviews flow diagram for the identified and included articles. The following research designs were employed for the 93 included studies: longitudinal-retrospective (*n* = 41, 44%); survey/cross-sectional design (*n* = 39, 42%); interview/qualitative research design (*n* = 5, 5%); longitudinal-prospective (*n* = 3, 3%); mixed-methods design (*n* = 3, 3%), and case–control research design (*n* = 2, 2%). The majority of the studies were conducted in the USA (*n* = 32, 34%), along with Italy (*n* = 7, 8%); Turkey (*n* = 6, 6%); Canada (*n* = 5, 5%); and the Netherlands (*n* = 5, 5%). The remaining studies were conducted in China, Taiwan, South Korea, the UK, Iran, Brazil, France, India, Austria, Croatia, Ireland, Mexico, Germany, Denmark, Israel, Japan, Scotland, and pan-European. The average sample size was 23 371, and the age across all studies ranged from 19 to 101 years.

### Impact of COVID-19 on BC services (Table 2)

Overall, 82 articles addressed the impact of COVID-19 on BC services (Research question 1), and narratives from these articles were organized by sub-themes: screening and diagnostic services; treatment (e.g. surgery, chemotherapy, and/or radiation therapy) and follow-up care (e.g. routine visits, examinations, and scans/tests) [107]; and telemedicine.

#### Screening and diagnostic services

Forty articles identified in the review addressed screening and diagnostic services during COVID-19 (Table 2). BC clinics experienced a reduction in varying breast screening procedures [23, 27, 28, 38, 45, 49] resulting in a lower number of patients presenting to breast clinics during the pandemic [24]. Patients reported delays in BC screening services [35]. There was a reduction in screening mammograms during the pandemic [18, 46, 47], especially with the onset of COVID-19 when screening programmes were closed [37, 55] and during times with high COVID-19 cases [22]. This reduction was similar for diagnostic mammograms [40, 42, 43, 53], as well as breast biopsies during the pandemic, resulting in fewer BC cases especially among early-stage diagnoses [21]. However, one study found that the methods of detection (i.e. breast imaging versus physical symptoms) during COVID-19 were similar to that of pre-COVID-19 times [50].

Another reason for the reduction in screening appointments was self-cancellations and missed appointments [17]; women were motivated to be screened but balanced the risk of contracting COVID-19 versus the risk of developing BC [32]. For those who did attend in-person screening appointments, women felt safe [41] and the efficacy of those visits was greater than that of pre-pandemic times [20,24]. Several articles addressed the recovery of cancelled and/or missed appointments following COVID-19 lockdown phases [25, 42, 44, 47, 48, 56]. However, the consequence of accumulated reduction in appointments may impact the diagnostic stage for women [43].

According to nine studies, a decline in the incidence of BC was noted from eight different countries [26, 30, 31, 33, 34, 39, 48, 52, 54]. No studies found contradictory results. While there was a reduction in overall BC cases during 2020, there were short-term increases in diagnoses in between lockdown phases [34, 36]. Late-stage diagnoses increased during the pandemic, but early-stage diagnoses decreased during the pandemic [21, 51]. Many women who received a diagnosis of BC were symptomatic [33, 34, 36, 51]; therefore, urgent and symptomatic cases were prioritized during the pandemic [19, 29, 34, 42].

#### Treatment and follow-up care

Of the 41 articles addressing treatment and follow-up care for BC (Table 2), many countries experienced a drop in in-person visits for services such as surgery, oncology, radiotherapy, and follow-up care (e.g. routine care and rehabilitation) [61, 64–66, 71, 83, 86, 87]. Many studies quantified treatment and appointment delays [70]. On average, the majority of women indicated a delay or change to their regular BC care during the pandemic [5, 59, 77, 78, 80, 85], and women expressed concerns about such delays on their cancer prognosis [6, 60, 82, 84]. Disruption to appointments and treatments was the result of either the health system [7, 71, 81] or by choice from the patient [76, 79].

The number of BC surgeries declined during the pandemic for a variety of reasons, such as a decrease in referrals, discontinuation in screening programmes, and safety precaution [49, 63, 71]. Women still experienced delays even after such procedures resumed [57]; however, the types of breast surgery performed during the pandemic did not differ greatly compared to 1-year prior [50]. Additional surgical guidelines and practices include the avoidance of reconstruction surgery, modified mastectomies, shortened length of hospital stay, and outpatient utilization [53, 70, 72]. With proper prioritization, breast surgery can be safely performed during COVID-19 [68, 72]. Several studies found the time to treatment, including surgery, to be longer [49, 54, 75]; however, one study found the wait time to surgery was lower than pre-pandemic times [58].

There was significant reduction in oncology treatments [73, 75]. Decreases in monotherapy administration and increases in oral anti-cancer drugs were common treatment alterations [62]. Moreover, radiation starts were lower during the pandemic, and modification of radiation therapy included regional nodal irradiation and adaptation of radiation therapy [67]. The reasons for delays included screening regulations on availability and accessibility to radiotherapy during the pandemic [69]. Hormonal treatment was more common during the pandemic, while chemotherapy was less common [52,61]. Specifically, one study found an increase in neoadjuvant endocrine therapy [49], and another study found that women treated during COVID-19 were more likely to receive neoadjuvant endocrine therapy [50]. While one study found no difference in the time interval from diagnosis to treatment [51], another study found the time from treatment to surgery increased [74]. Clinicians do not believe that treatment changes should be continued moving forwards from COVID-19 [55].

#### Telemedicine

Eleven articles included in the review addressed the adaptation of telemedicine for BC services (Table 2). Telemedicine was common for BC management during the pandemic for follow-up appointments, screening for benign disease, second opinion, and general guidance [85], and it proved to be an efficient way to safely monitor BC patients during the pandemic [93]. Even when cancellations recovered after lockdown phases, the use of electronic portals continued to increase, indicating the prolonged uptake of telemedicine among BC patients [56]. Telemedicine was widely accepted by BC patients [91, 92, 94] and clinicians [55] during the pandemic, and this satisfaction was slightly lower for surgical oncology visits [90]. Women identified telemedicine as a positive alternative to rehabilitation appointments when centres were closed during COVID-19 [7]. Women expressed higher satisfaction for video consultations compared to telephone [88]; however, most would have preferred in-person appointments [89].

### Impact of COVID-19 on health outcomes and well-being in women with BC (Table 3)

Overall, 46 articles addressed the impact of COVID-19 on health and well-being (Research question 2), and narratives from these articles were organized by sub-themes: clinical outcomes, QoL, and psychosocial well-being.

**Table 3.** The impact of COVID-19 on health outcomes and well-being (Research question 2).

Primary author, year	Study design	Country	Participant characteristics	Recruitment method/sample size	Time period, phase of lockdown	Results	Sub-themes (type of service)
Health outcomes and well-being (clinical outcomes, QoL, and psychosocial well-being)							
Bessa, 2021 [19]	Longitudinal (retrospective)	Brazil	Women who received mammograms (i) Female only (ii) Age range 50–69 years	*n* = 1 939 415 (pre-COVID) *n* = 1 126 688 (COVID)	2019/2020	Women reported more palpable lumps with screening during the pandemic year	Clinical outcomes (screening and diagnostic services)
Bonadio, 2021 [20]	Longitudinal (retrospective)	Brazil	Patients with a diagnosis of BC	*n* = 457 (pre-COVID) *n* = 268 (COVID)	September–January 2019–20/2020–21	More advanced stages of BC were detected during the pandemic and women who were diagnosed during the pandemic reported more symptoms	Clinical outcomes (screening and diagnostic services)
Eijkelboom, 2021 [52]	Longitudinal (retrospective)	The Netherlands	Women with a recent diagnosis of BC (i) Similar age distribution between years	*n* = 5685 (2018–pre-COVID) *n* = 5838 (2019–pre-COVID) *n* = 4769 (2020–COVID)	Weeks 2 through 17 of 2018/2019/2020	Screen-detected tumours decreased and remained low during 2020, yet non-screen-detected tumours in 2020 recovered from initial decrease	Clinical outcomes (screening and diagnostic services)
Eijkelboom, 2021 [26]	Longitudinal (retrospective)	The Netherlands	Women with a diagnosis of BC (i) 50–74 years of age	*n* = 5306 (2020–COVID) *n* = 7302 (2019–COVID) *n* = 7250 (2018–COVID)	Weeks 2 through 35 of 2020/2019/2018	There was a larger decrease in screen-detected compared to non-screen-detected tumours during the pandemic year compared to 2018/2019	Clinical outcomes (screening and diagnostic services)
Kaltofen, 2020 [30]	Longitudinal (retrospective)	Germany	BC patients (i) Median age: 54.7 years (COVID) (ii) Median age: 56.6 years (pre-COVID)	*n* = 327 (2020–COVID) *n* = 365 (2019–pre-COVID)	January–June 2020/2019	High-stage and symptomatic diagnoses were more common during the pandemic, especially during lockdown phases	Clinical outcomes (screening and diagnostic services)
Kang, 2021 [31]	Longitudinal (retrospective)	South Korea	Patients receiving BC screening services and surgical treatment (i) Median age: 53 years	*n* = 1369 (2020–COVID) *n* = 1669 (2019–pre-COVID)	February–July 2020/2019	Higher stages of cancer were diagnosed in the later period of the pandemic year	Clinical outcomes (screening and diagnostic services)
Kiziltan, 2021 [33]	Longitudinal (retrospective)	Turkey	BC patients (i) Female only (ii) Age range 20–82 years (median age: 51)	*n* = 250 (pre-COVID) *n* = 146 (COVID)	Pre-pandemic versus March–June 2020	Patients had larger and more lymph node metastases during the pandemic and more early-stage cancer were diagnosed pre-pandemic	Clinical outcomes (screening and diagnostic services)
Knoll, 2021 [34]	Longitudinal (retrospective)	Austria	Newly diagnosed BC and gynaecological cancer patients (i) Median age = 63 years (pre-COVID) (ii) Median age = 60 years (COVID)	*n* = 495 (pre-COVID) *n* = 394 (COVID) Breast only (both time points): *n* = 596	March–December 2019/2020	More women reported tumour-associated symptoms, and those BC patients were more likely to be referred in from a specialist for screening during the pandemic	Clinical outcomes (screening and diagnostic services)
Koca, 2021 [53]	Longitudinal (retrospective)	Turkey	Patients with a BC diagnosis (i) 146 women, 2 men (ii) Age range: 22–91 years; median age: 51.2 years	Overall: *n* = 148 *n* = 70 (pre-COVID) *n* = 78 (COVID)	2019/2020	Tumour size and axillary lymph node positivity diagnoses were higher in 2020 than in 2019	Clinical outcomes (screening and diagnostic services)
Linck, 2021 [36]	Longitudinal (retrospective)	France	BC diagnosis (i) Pre-COVID mean age: 66 years (ii) COVID mean age: 64 years	*n* = 120 (pre-COVID) *n* = 134 (COVID)	2019/2020	75% of diagnoses during the pandemic were symptomatic which is higher than during non-pandemic. Tumour size was larger during post-pandemic period compared to 2019, and metastasis was more likely in BC diagnoses during 2020	Clinical outcomes (screening and diagnostic services)
Romics, 2021 [72]	Longitudinal (prospective)	Scotland	BC patients undergoing surgery (i) Median age: 54 years	*n* = 179	March–May 2020	Tumour size was larger during COVID-19, and ER-negative and HER2-positive diagnoses were significantly higher	Clinical outcomes (screening and diagnostic services)
Syed, 2020 [74]	Case–control	India	BC patients on neoadjuvant therapy awaiting surgery (i) Mean age: 45.35 ± 10.6 years	*n* = 54	March–August 2020	Delayed neoadjuvant chemotherapy caused disease progression	Clinical outcomes (treatment and follow-up care)
Tonneson, 2021 [50]	Longitudinal (retrospective)	The USA	BC patients with a new diagnosis (i) Pre-COVID 373 females/3 males Mean age: 60.5 years (ii) COVID Female only Mean age: 59.8 years	*n* = 376 (pre-COVID) *n* = 197 (COVID)	March–August 2019/2020	Stage of diagnosis between 2 years did not differ greatly	Clinical outcomes (screening and diagnostic services)
Toss, 2021 [51]	Longitudinal (retrospective)	Italy	Patients diagnosed with BC (i) Pre-COVID 221 females/2 males (ii) COVID 174 females/3 males	*n* = 223 (2019) *n* = 177 (2020)	May–July 2019/2020	The number of BC diagnoses with metastatic lymph nodes significantly increased in 2020. Early-stage diagnoses decreased from 2019 to 2020, whereas late-stage diagnoses increased from 2019 to 2020	Clinical outcomes (screening and diagnostic services)
Vanni, 2020 [75]	Longitudinal (retrospective)	Italy	BC patients awaiting surgery (i) Pre-COVID median age: 62 (ii) COVID median age: 60.8	*n* = 209 (pre-COVID) *n* = 223 (COVID)	March–May 2019/2020	There was an increase in lymph node involvement at diagnosis during COVID	Clinical outcomes (screening and diagnostic services)
Wadasadawala, 2021 [95]	Cross-sectional	India	BC patients undergoing treatment (i) Median age: 45 years	*n* = 138	March–June 2020	The average expenditure for BC women was 32% higher during the pandemic, yet the average household income decreased by 25%. More so, more than 65% of women had no income during the lockdown, which caused financial distress. This in turn impacted access to food and medicine	QoL (treatment and follow-up care)
Bargon, 2021 [96]	Cross-sectional	The Netherlands	Patients with BC (i) 99.4% females and 0.6% males (ii) Mean (SD) age: 56 (9.8) years	*n* = 1051	April 2020	Due to COVID barriers, one-third of patients were less likely to contact their general practitioner (GP). Emotional functioning and loneliness worsened significantly during COVID-19, and there were slight increases in patient-reported outcomes for physical, QoL, and role functioning	QoL Psychosocial well-being (treatment and follow-up care)
Helm, 2020 [7]	Cross-sectional	The USA	BC survivors (i) Female only (ii) Mean age: 55.3 ± 9.8 years	*n* = 15	May 2020	Distress was higher at the time of rehab closure compared to reopening. At the time of closure, distress was correlated with reduced physical activity. At the time of reopening, reduced distress was correlated with reduced fatigue	QoL Psychosocial well-being (treatment and follow-up care)
Ludwigson, 2022 [91]	Mixed-methods	The USA	BC patients (i) Majority over the age of 45 years	*n* = 133 (survey) *n* = 20 (interview)	August 2020–February 2021	50% of women reported concerns over COVID-19 specific to their BC care, yet only 18% of women reported BC disruption. Many women endorsed anxiety and/or depression during the pandemic, and 22% reported a decrease in income due to COVID-19	QoL Psychosocial well-being (treatment and follow-up care)
Massicotte, 2021 [97]	Cross-sectional	Canada	BC patients undergoing treatment (i) Female only (ii) Mean age: 53.6 ± 10.9 years	*n* = 36	April–May 2020	Psychological impact, including anxiety, depression, insomnia, and fear of cancer recurrence, was correlated with stressors such as difficulty obtaining food, medicine, and essentials, postponement or cancellation of cancer treatment, changes in cancer care trajectory, and postponement of medical tests	QoL Psychosocial well-being (treatment and follow-up care)
Zhang, 2021 [82]	Qualitative	The USA	Women participating in online forums within Breastcancer.org, a large BC support community in the USA	Unrestricted, volunteer sampling, *n* = 192	March–April 2020	Topics discussed included stress and concerns about COVID-19, coping and support, work and financial changes, educational information, and COVID-19 susceptibility due to cancer	QoL Psychosocial well-being (treatment and follow-up care)
Zhao, 2021 [83]	Cross-sectional	The USA	BC patients (i) Mean age (White): 61.6 ± 11.6 years (ii) Mean age (Black): 63.6 ± 11.9 years	*n* = 1300	July–September 2020	Patients reported greater isolation during the pandemic compared to pre-pandemic and one-third of respondents expressed financial difficulties during the pandemic	QoL Psychosocial well-being (treatment and follow-up care)
Alipour, 2020 [81]	Cross-sectional	Iran	Patients who were scheduled to have a BC appointment but did not attend their visit (i) Female (ii) Age range 19–64 years (mean age: 42.9)	Random selection of 10% of patient list *n* = 134	March–May 2020	The majority of women indicated the cause of delay to be fear of COVID-19 transmission in the breast clinic. Breast or emotional pain did not significantly increase during COVID-19	QoL Psychosocial well-being (treatment and follow-up care)
Seven, 2021 [87]	Qualitative	Turkey	BC survivors who completed treatment within the last 5 years (i) Female only (ii) Mean age: 51 ± 5.9 years	Convenience sample, *n* = 18	May 2020	Women expressed physical functioning, role functioning, emotional functioning, cognitive functioning, and social functioning, including fatigue; sleep problems, impaired physical functioning, pain, physical symptoms (e.g. lymphoedema), anxiety, and depression	QoL Psychosocial well-being (treatment and follow-up care)
Bizot, 2021 [88]	Cross-sectional	France and Italy	Patients with BC (i) Majority (72.6%) aged ≥50 years	*n* = 1299	April–May 2020	Patients with telehealth services did not experience much anxiety and patients reported satisfaction with telehealth services. 52.6% of participants had low/no anxiety	Psychosocial well-being (treatment and follow-up care; telemedicine)
Chen, 2021 [59]	Cross-sectional	China	Patients with BC receiving active treatment (i) Majority (65.1%) aged ≥46 years	*n* = 834	March 2020	Depression, anxiety, and insomnia were expressed by women with BC, and this psychological impact was associated with factors such as affected treatment, living alone, comorbidities, and deterioration of BC	Psychosocial well-being (treatment and follow-up care)
Choobin, 2021 [60]	Cross-sectional	Iran	BC patients with a primary diagnosis of BC in Iran (i) Female only (ii) Mean age: 45.9 ± 9.6 years	Volunteer recruitment, *n* = 139	May 2020	About 30% of women indicated delayed BC services and those women expressed higher emotional vulnerability and worry to COVID-19	Psychosocial well-being (treatment and follow-up care)
Cui, 2020 [98]	Cross-sectional	China	BC patients and nurses (i) Majority age of BC patients under 55 (72%)	*n* = 207 patients *n* = 684 nurses	February 2020	BC patients indicated high levels of depression, anxiety, insomnia, and post-traumatic stress disorder (PTSD) during this time	Psychosocial well-being (treatment and follow-up care)
Hamlish, 2021 [80]	Cross-sectional	The USA	BC survivors (i) Mean age: 47.9 ± 10.9 years (ii) Race: 81% White/19% Black	Snowball sampling via social media and email, *n* = 570	April 2020	Distress or higher level of worry towards cancer care was common during the pandemic	Psychosocial well-being (treatment and follow-up care)
Kennard, 2021 [85]	Longitudinal (prospective)	The USA	BC patients receiving active treatment (i) Mean age: 60.6 ± 13.3 years	*n* = 73	March–June 2020	56% of patients had no change in treatment, while 44% did experience change in recommended treatment. The two groups showed no difference in anxiety/depression	Psychosocial well-being (treatment and follow-up care)
Kim, 2021 [84]	Cross-sectional	South Korea	Patients diagnosed with BC within the last 2 years (i) Age: 42.8 ± 7.7 years	*n = *154	April–June 2020	Women reported moderate-to-severe levels of depression and anxiety, and fear of cancer recurrence was high. The only psychological measure associated with treatment changes was depression	Psychosocial well-being (treatment and follow-up care)
Kirkegaard, 2021 [32]	Qualitative	Denmark	Women eligible for BC screening (i) Female only (ii) Mean age: 62 years	Convenience sample, *n* = 33	April 2020	Women who postponed/cancelled their mammography were motivated to be screened, but balanced the risk of COVID-19 versus the risk of BC in deciding whether to attend mammography	Psychosocial well-being (screening and diagnostic services)
Koral, 2021 [99]	Cross-sectional	Turkey	BC survivors with mean age: 43.2 ± 4.9 years	*n* = 82	May 2020	Many women reported high fear of cancer recurrence, yet women with high spiritual well-being and psychological resilience experienced less fear of cancer recurrence	Psychosocial well-being (treatment and follow-up care)
Li, 2020 [100]	Cross-sectional	China	BC patients and survivors of early-stage BC (i) Majority younger than 55 years (78.4%)	*n = *658	February 2020	Women were more likely to express extreme levels of depression, anxiety, insomnia, and distress with exposure to Wuhan, poor general condition, treatment discontinuation or disruption, and those with metastatic disease	Psychosocial well-being (treatment and follow-up care)
Magno, 2020 [101]	Cross-sectional	Italy	Patients with a new diagnosis of BC awaiting surgery (i) Female only (ii) Mean age 56.3 years	*n = *86	March 2020	The majority of women expressed heightened emotional distress during the pandemic; women expressed exacerbated cancer fears during the pandemic and additional fear specific to COVID-19. Additionally, women expressed concern over delays for cancer treatment, and they expressed a sense of vulnerability of having cancer during the pandemic	Psychosocial well-being (treatment and follow-up care)
Papautsky, 2021 [102]	Cross-sectional	The USA	BC survivors (i) 632 females/1 male (ii) Mean age: 47.93 ± 10.95 years	*n* = 633	April–May 2020	The pandemic has caused worry for many BC survivors, and this worry was more extreme for vulnerable individuals, such as those in active treatment, immunocompromised, and experiencing delays in care	Psychosocial well-being (treatment and follow-up care)
Rentscher, 2021 [103]	Case–control	USA	Case: non-metastatic BC survivors (i) Mean age: 67.9 ± 5.3 years	Total: 427 Case: *n* = 262 Control: *n* = 165	May–September 2020	Both BC patients and non-cancer controls expressed an increase in anxiety and depression during COVID-19. Both groups showed a similar increase in loneliness during the pandemic, as well	Psychosocial well-being (treatment and follow-up care)
Savard, 2021 [6]	Qualitative	Canada	Women with non-metastatic BC who were scheduled to have chemotherapy (i) Female only (ii) Mean age: 51.8 years	Volunteer sample *n* = 23	May 2020	Women expressed concerns such as treatment-related immunosuppression, higher risk of catching COVID-19 in the hospital, distress related to going to treatment alone, increased responsibility at home, social isolation and decreased family relationships; coping strategies used, and difficulty receiving professional mental health services and social support	Psychosocial well-being (treatment and follow-up care)
Schifferdecker, 2021 [41]	Qualitative	The USA	Women eligible for mammography, with and without a BC diagnosis (i) Mean age: 57 years	*n* = 30 (*n* = 22 without BC, *n *= 8 with BC)	March–August 2020	Women described the following decision factors for attending screening appointments: balancing the risk of cancer versus COVID-19, fear of testing, safety of testing, and general anxiety	Psychosocial well-being (screening and diagnostic services)
Shinan-Altman, 2020 [79]	Cross-sectional	Israel	BC patients (i) Female only (ii) Mean age: 51 years	*n* = 151	April 2020	Contact with health-care providers was relatively low, but for women with perceived low health status, mastery, and high anxiety had more contact with health professionals. On the contrary, women were likely to cancel an appointment if they did not perceive their health as bad, if they had a lower sense of mastery, and a low level of social support. Patients who cancelled appointments for oncology/haematology had high anxiety/vulnerability	Psychosocial well-being (treatment and follow-up care)
Soriano, 2021 [57]	Cross-sectional	The USA	Patients with a diagnosis of BC in the USA with postponed surgery (i) Female only (ii) Mean age: 60.1 ± 13.2 years	*n* = 50	June–August 2020	Women reported mild psychosocial well-being while awaiting surgery and/or directly after postponed surgery. Of these women, 26% indicated a strong fear of cancer progression with postponed surgery. Of the women still awaiting surgery, there was a poorer indication of satisfaction and communication with their BC care team	Psychosocial well-being (treatment and follow-up care)
Swainston, 2020 [73]	Cross-sectional	UK	Patients diagnosed with primary BC (i) Female only (ii) Mean age: 51 years	Volunteer sampling via support platforms, *n* = 234	2020	Women who experienced a service disruption showed greater levels of general emotional vulnerability and COVID-specific vulnerability, which in turn impacted distress such as anxiety, depression, and impaired cognitive function	Psychosocial well-being (treatment and follow-up care)
van der Molen, 2021 [104]	Cross-sectional	The Netherlands	Patients and survivors of BC (i) 1045 females/6 males (ii) Mean age: 56 ± 9.9 years	*n* = 1051	April 2020	27% of respondents reported clinically relevant anxiety/depression. Those participants reported higher barriers to contacting their GP and BC care team and more disruption to their BC treatment	Psychosocial well-being (treatment and follow-up care)
Vanni, 2020 [76]	Longitudinal (retrospective)	Italy	Patients attending breast screening centre (i) Suspicious breast lesion (a) Pre-COVID mean age: 57.6 years (b) COVID mean age: 59.5 years (ii) BC diagnosis (a) Pre-COVID mean age: 64.3 years (b) COVID mean age: 61.2 years	*n* = 82 (suspicious breast lesion) *n* = 78 (BC group)	January–March 2020	Women from both clinical groups were more likely to refuse BC procedures post-COVID-19 due to risk of COVID-19 infection	Psychosocial well-being (screening and diagnostic services)
Yasin, 2021 [105]	Cross-sectional	Turkey	Patients with BC (i) Female only (ii) Mean age: 53.2 ± 10.79 years	*n* = 298	April–August 2020	The majority of women expressed heightened anxiety during COVID-19, and this was significantly higher among women with a metastatic diagnosis of cancer. However, after controlling for demographic variables, there were no significant differences between the two groups	Psychosocial well-being (treatment and follow-up care)
Yousefi Afrashteh, 2021 [106]	Cross-sectional	Iran	BC patients (i) Female only (ii) Mean age: 38.97 ± 12.37 years	*n* = 210	September–November 2020	Depression and anxiety were positively associated with death anxiety and negatively associated with self-compassion. Self-compassion was found to be a mediating factor for psychological well-being on death anxiety	Psychosocial well-being (treatment and follow-up care)

#### Clinical outcomes

Fifteen studies discussed the impact of disrupted BC services on clinical outcomes (e.g. late-stage or change in diagnosis) (Table 3). Despite the decrease in the overall incidence of BC during lockdown phases, late-stage diagnoses increased, indicating more symptomatic and progressed cancer diagnoses [20, 30, 31]. Even more, the number of BC diagnoses with metastatic lymph nodes significantly increased in 2020, and women reported more tumour-associated symptoms and palpable lumps [19, 33, 34, 51]; researchers predict an increase in BC mortality as a result [36, 53, 75]. ER-negative and HER2-positive rate was significantly higher during the pandemic [72], and delayed neoadjuvant therapy caused disease progression and delayed surgery may be associated with worsened BC, such as lesions [74]. Women with minimal symptoms were more likely to experience a delay in BC screening; there was a larger decrease in screen-detected tumours and diagnoses during the pandemic [26, 52]. On the contrary, one study found that BC stage at diagnosis did not differ significantly from pre-COVID-19 to COVID-19 [50].

#### QoL

Nine studies addressed the QoL of women with BC during the pandemic (Table 3), including physical functioning, social functioning, emotional functioning, and cognitive functioning [87, 96]. Fatigue and physical inactivity were mentioned as a consequence of rehabilitation centre closures during the pandemic [7]. Women expressed an increase in responsibility at home and a decrease in social support [97]. Furthermore, women experienced financial distress due to a decrease in household income and an increase in expenditure [82, 83, 91], which can impact access to food and medicine [95]. Several studies conducted early into the pandemic did not find worsened QoL [81, 96]; however, the results may not represent any long-term consequences of the pandemic on women with BC.

#### Psychosocial well-being

Many studies (*n* = 30) investigated the impact of COVID-19 on psychological, emotional, and social well-being (Table 3). Disruption to BC care caused treatment-related stress and fear [6, 7, 80, 82, 97]. BC patients with heightened depression and anxiety expressed a higher sense of death anxiety [106] and BC recurrence [57, 97, 101], and one study found that women with heightened anxiety were more likely to cancel their appointments and stay in contact with their BC care team remotely [79]. However, some women reported a higher difficulty to contact their BC care team during the pandemic [96]. Women who received telemedicine during the pandemic were less anxious as they felt assured with continued communication with their BC care team [88], and with telemedicine communication, treatment disruption did not worsen depression and anxiety for women [85].

BC patients expressed depression, along with anxiety and impaired cognitive function [41, 73, 84, 87, 91, 98, 105] during the pandemic, and such psychological impact can be associated with factors such as affected treatment, living alone, comorbidities, immunosuppression, undergoing active treatment, deterioration/metastasis of BC, lacking communication with GP and/or BC care team, and living in high-infection areas [59, 100, 102, 104, 105]. Many women expressed worry and anxiety about contracting COVID-19 [60, 76, 82, 91], including fear of COVID-19 transmission in BC facilities [81] and concerns about treatment-related immunosuppression [6]. Women balanced the risk of contracting COVID-19 versus the risk of developing BC [32, 41].

Women expressed worsened emotional functioning and vulnerability during the pandemic [60, 96, 101], and loneliness was another common consequence [6, 96, 103]. BC patients felt isolated during the pandemic [83], and loneliness was attributed to social isolation and attending treatment visits alone [6]. Insomnia and fatigue were also issues mentioned by women with BC [7, 59, 87, 97, 98, 100], and some BC patients experienced PTSD during the pandemic [98]. Many women discussed the importance of utilizing coping strategies [6, 82]; spirituality and psychological resilience improved patients’ fear of cancer recurrence [99].

### Variation in the impact of COVID-19 on BC by SDH (Table 4)

Overall, 26 articles assessed how the impact of COVID-19 on BC services and health outcomes and well-being differed depending on SDH, including age (*n* = 14); race/ethnicity (*n* = 10); insurance status (*n* = 7); region (*n* = 6); education (*n* = 2); marital status (*n* = 2); and income (*n* = 1).

#### BC services

There were racial differences with screening appointments (*n* = 6); Black, Hispanic, and Asian women were more likely to experience a decrease in screening services during the pandemic [17, 18, 25, 27, 43, 47]. Delays for BC treatment were longer for Black patients compared to White patients, yet this difference was apparent prior to pandemic as well [66]. Additionally, reduction in screening appointments was associated with age, low socio-economic status (SES), limited health insurance, area of residence, and facility type [17, 18, 25, 27, 31, 35, 37]. However, there were contradicting findings for the disruption of screening services based on health insurance status [38]. Additionally, women living in areas of high COVID-19 infection were also more likely to cancel/postpone their screening appointment [45].

For treatment and follow-up appointments (*n* = 11), women with limited ability to pay (i.e. public health insurance) were likely to experience treatment delays [78]. The following characteristics were associated with specific treatment alterations and delays: older age, marital status, and living in specific regions, such as high-infection areas [49, 62, 67, 68]. For follow-up appointments, younger BC survivors were likely to experience more delays than older BC survivors [5], and variables associated with those who did attend appointments included younger age and lower income [86]. Low educated BC patients were less satisfied with telemedicine [92], and while one study found that older women preferred telemedicine [89], another study found lower satisfaction of telemedicine among women older than 40 years [88].

#### Health outcomes and well-being

Fewer studies (*n* = 5) addressed the varying impact of COVID-19 on overall well-being (Table 4). White BC patients reported a higher level of worry than Black BC patients; however, Black BC patients were more likely to experience delays and disruptions prior to the pandemic [80]. Financial difficulties during COVID-19 were more common for minorities and for women with limited health insurance [83]. Likewise, younger age and living further from BC clinic were found to be associated with financial distress [95]. Older women expressed more anxiety due to minimal BC communication [88]; however, younger women expressed higher rumination and emotional vulnerability specific to COVID-19 [60].

**Table 4.** The varied impact of COVID-19 based on SDH (Research question 3).

Primary author, year	Study design	Country	Participant characteristics	Recruitment method/sample size	Time period, phase of lockdown	Results	Sub-themes (SDH)
*SDH*							
Amornsiripanitch, 2021 [17]	Longitudinal (retrospective)	The USA	All BC patients within a scheduling database (i) Female (ii) Age range 24–101 years (mean age 60.8)	*n* = 15 792 (pre-COVID) *n* = 16 595 (COVID)	June–August 2019/ March–June 2020/ June–August 2020	Women who cancelled their mammography during reopening were older and underserved (with Medicare and non-White). Relative risk (RR) of cancellation increased with age: 1.20 versus 1.27 versus 1.36 for ages at 25th, 50th, and 75th quartile (*P* < .001). RR of cancellation was higher for Medicare recipients: 1.41 versus 1.26 versus 1.21 for Medicare, Medicaid, and other insurances (*P* < .001). RR for cancellation was higher for minorities: 1.34 versus 1.25 for non-White women compared to White women (*P* = .03)	BC services (age, insurance, and race/ethnicity)
Amram, 2021 [18]	Longitudinal (retrospective)	The USA	Women who completed BC screening (i) Female (ii) Mean age: 62.4 ± 11.1 years	*n* = 55 678 (pre-COVID) *n* = 27 522 (COVID)	April–December 2019/2020	The reduction of screening services was greater among minorities compared to women who were White *(P < *.001). The reduction was also greater for women living in rural areas (*P* < .001) and for women without health insurance/with Medicaid (*P* < .001)	BC services (race/ethnicity, region, and insurance)
Bruce, 2021 [89]	Mixed-methods	The UK	BC patients with a scheduled telephone consultation, who had undergone breast surgery (i) Mean age: 61.5 ± 12.0 years	Random selection, *n* = 30	n/a	A higher proportion of younger women preferred face-to-face appointments over telemedicine (73%) compared to older women (37%)	BC services (age)
DeGroff, 2021 [25]	Longitudinal (retrospective)	The USA	Women receiving screening tests within national programme providing early detection for women of without health insurance	*n* = 487 645	January–June 2015–20	The decline in screening services was greater for American Indian/Alaskan Indian women (98% decline). Screening services recovered by June 2020, except for women living in rural areas; for these women, screening rates remained >50% below the 5-year average	BC services (race/ethnicity and region)
Fedele, 2021 [62]	Cross-sectional	Italy	Oncologists who provided treatment to women with metastatic BC	*n* = 90	May–June 2020	Most oncologists (80%) were likely to delay chemotherapy treatment for older women	BC services (age)
Fedewa, 2021 [27]	Longitudinal (retrospective)	The USA	Screening rates for women receiving BC screening among community health centres (i) 50–74 years of age	49.6% (2020–COVID) 53.9% (2019) 45.8% (2018)	July 2020/ July 2019/ June 2018	The decline in screening rates was greater among Black patients [screening rate ratio (SRR): 0.88; 95% CI: 0.87–0.90] and lower for uninsured patients (SRR: 0.85; 95% CI: 0.84–0.86)	BC services (race/ethnicity and insurance)
Hawrot, 2021 [66]	Longitudinal (retrospective)	USA	Newly diagnosed, early-stage BC patients (i) Majority 50–70 years of age	*n* = 164 (2020–COVID) *n* = 202 (2018–COVID)	January–May 2020/2018	The time-to-treatment initiation for Black patients was 16 days slower than for White patients on average (95% CI: 6.9–24.6, *P* = .001)	BC services (race/ethnicity)
Kang, 2021 [31]	Longitudinal (retrospective)	South Korea	Patients receiving BC screening services and surgical treatment (i) Median age: 53 years	*n* = 1369 (2020–COVID) *n* = 1669 (2019–pre-COVID)	February–July 2020/2019	For younger women (<65 years), there was a significant difference in cancer stage at diagnosis (*P = *.002)	BC services (age)
Koch, 2020 [67]	Longitudinal (retrospective)	Canada	Number of radiation therapy starts	*n* = 160 (pre-COVID) *n* = 118 (COVID)	March–April 2019/2020	A smaller proportion of patients older than 50 years received radiation therapy during 2020 compared to 2019 (*P* = .0027)	BC services (age)
Lee, 2020 [68]	Longitudinal (retrospective)	South Korea	Newly diagnosed BC patients scheduled for surgery (i) 61 females and 1 male (ii) Mean age: 55.7 ± 11.2 years	*n* = 62	February–April 2020	Patients who did not experience surgical delays were younger (*P* = .003) and single (*P = *.038)	BC services (age and marital status)
Li, 2021 [35]	Cross-sectional	The USA	Women attending BC screening (i) Majority in range 40–70 years	*n* = 570	December 2020–January 2021	Women who reported a delay in screening appointment tended to be younger (*P* = .014)	BC services (age)
Miller, 2021 [37]	Longitudinal (retrospective)	The USA	Patients receiving screening mammograms (i) Pre-COVID: median age 62 years (ii) COVID: median age 62 years	*n* = 10 757 (pre-COVID) *n* = 9062 (COVID)	March–October 2019/2020	A lower likelihood of non-screening included younger age [odds ratio (OR): 0.78, *P* < .001]; living in a higher poverty area (OR: 0.991, *P* = .014); lack of health insurance (OR: 0.65, *P* = .007); and longer travel time (OR: 0.998, *P* < .001)	BC services (age, region, and insurance)
Mo, 2021 [86]	Longitudinal (retrospective)	The USA	Early-stage BC survivors 2812 females/18 males	*n* = 2830	2019–20	Variables associated with those who did attend a follow-up visit after included younger age (*P* = .049) and lower household income (*P* = .031)	BC services (age and income)
Nyante, 2021 [38]	Longitudinal (retrospective)	The USA	Women receiving BC screening services (i) Mean age: 59 ± 2 years	*n* = 39 444	January 2019 to 2 March 2022/3 March 2020 to 30 September 2020	After the onset of COVID-19, women without insurance were prioritized for mammograms [predicted probability (PP): 16; 95% CI: 14–17; *P* < .001] and for biopsies (PP: 27; 95% CI: 23–32; *P* < .001)	BC services (insurance)
Papautsky, 2020 [5]	Cross-sectional	The USA	BC survivors (i) 608 females/1 male (ii) Mean age: 47.8 years	Snowball sampling via social media, *n* = 609	April 2020	Younger BC survivors were likely to experience more delays than older BC survivors (95% CI: 95–99, *P* < .001)	BC services (age)
Picardo, 2021 [92]	Cross-sectional	Italy	BC and gynaecological cancer patients receiving follow-up visits via telemedicine (i) Female only (ii) Median age of BC group: 67 years	*n* = 345 (*n* = 267 BC, *n* = 78 gynaecological cancer)	March–May 2020	Less educated BC patients were more likely to express enhanced care than more educated BC patients (*P* = .034), but women of lower education were less satisfied with telemedicine than more educated women (*P* < .001)	BC services (education)
Satish, 2021 [78]	Cross-sectional	The USA	BC patients (i) 348 females/2 males (ii) Mean age: 57.1 ± 14 years	*n* = 350	February–April 2020	Women with Medicaid (public health insurance) were more likely to experience treatment delays/changes (OR: 3.04; 95% CI: 1.32–7.27; *P* = .01)	BC services (insurance)
Sprague, 2021 [43]	Longitudinal (prospective)	The USA	Screening and diagnostic mammograms (i) Female only	*n* = 461 083 screening mammograms *n* = 112 207 diagnostic mammograms	January 2019–July 2020	Recovery for mammogram appointments was lower among Hispanic women (64.9%; 95% CI: 48.5%–86.9%) and Asian women (74.8%; 95% CI: 61.1%–91.5%) compared to White women (102.5%; 95% CI: 91.6%–114.7%) and Black women (103.0%; 95% CI: 90.4%–117.4%)	BC services (race/ethnicity)
Toyoda, 2021 [45]	Cross-sectional	Japan	Women with a scheduled screening appointment (i) Age range 30–79 years	*n* = 1874	April–May 2020	Women living in areas of Japan with a declared state of emergency were more likely cancel/postpone their appointment (prevalence ratio: 1.25; 95% CI: 1.08–1.46; *P* < .05)	BC services (region)
Velazquez, 2021 [47]	Cross-sectional	The USA	Performed mammograms	*n* = 9291	January 2019–January 2021	Black and Latina women were more likely to experience a decrease in mammograms (*P* < .05)	BC services (race/ethnicity)
Wilke, 2021 [49]	Cross-sectional	The USA	BC patients (on behalf of breast surgeons) (i) Mean age: 62.7 years	*n* = 2791	March 2020–March 2021	Women were more likely to receive neoadjuvant endocrine therapy before surgery/usual practice with older age (OR: 1.1; *P* < .05) and in specific regions (northeast/southeast) (OR: 2.3/1.7; *P* < .05)	BC services (age and region)
Bizot, 2021 [88]	Cross-sectional	France and Italy	Patients with BC (i) Majority aged ≥50 years	*n* = 1299	April–May 2020	Satisfaction with telemedicine was correlated with age, with lower satisfaction for women 40–49 (OR: 2.2; 95% CI: 1.1–4.3; *P* < .27) and women 50–50 years (OR: 2.3; 95% CI: 1.2–4.4; *P* = .017) compared to women younger than 40 years	BC services Health and well-being (age)
Choobin, 2021 [60]	Cross-sectional	Iran	BC patients with a primary diagnosis of BC in Iran (i) Female only (ii) Mean age: 45.9 ± 9.6 years	Volunteer recruitment, *n* = 139	May 2020	Younger women expressed higher rumination (*P* = .05) and emotional vulnerability specific to COVID (*P* = .01) compared to older women	Health and well-being (age)
Hamlish, 2021 [80]	Cross-sectional	The USA	BC survivors (i) Mean age: 47.92 ± 10.86 years (ii) Race: 81% White/19% Black	Snowball sampling via social media and email, *n* = 570	April 2020	White respondents addressed a higher level of worry specific to cancer (*P* < .001) and all-health worry (*P* < .001) than Black respondents, but this difference may be attributed to previous experience to treatment delays and disruptions in care, as Black women experienced more delays and treatments prior to the pandemic	Health and well-being (race/ethnicity)
Wadasadawala, 2021 [95]	Cross-sectional	India	BC patients undergoing treatment (i) Median age: 45 years	*n* = 138	March–June 2020	Financial distress was slightly lower for older women (OR: 0.43; 95% CI: 0.17–1.11; *P* < .1) and higher for women living outside Maharashtra (treatment location) (OR: 2.46; 95% CI: 0.86–7.00; *P* < .1)	Health and well-being (age and region)
Zhao, 2021 [83]	Cross-sectional	The USA	BC patients (i) Mean age (White): 61.6 ± 11.6 years (ii) Mean age (Black): 63.6 ± 11.9 years	*n* = 1300	July–September 2020	From analysis of White and Black respondents, the only significant difference concerned financial difficulties: Medicaid recipients (80% of which are Black) experienced more financial difficulties during COVID-19 (*P* < .001)	Health and well-being (race/ethnicity and insurance)

## Discussion

### Statement of principal findings

There has been a substantial impact on BC services and health outcomes and well-being during COVID-19 occurring across the BC care continuum, from screening services to follow-up care. Therefore, women with a prior diagnosis of BC or awaiting a diagnosis of BC were at risk of disrupted health care and experienced worsened health outcomes during the pandemic, which is comparable to research conducted on all cancer types during the pandemic [108].

### Interpretation within the context of the wider literature

The implementation of a triage system helped prioritize high-risk BC patients [109], yet the prioritization for high-risk patients may negatively impact the future prognosis of low-risk patients, which is consistent with overall cancer research during the pandemic [110]. The overall decrease in BC incidence may be the result of BC screening programmes closures [52], and due to limited capacity and safety precautions, there was also a reduction of in-person appointments for treatment and follow-up care [50, 72].

There was a negative impact on QoL and psychosocial well-being which is consistent with general research regarding COVID-19 and cancer [111]. The combination of living with BC during COVID-19 may exacerbate psychosocial distress and death anxiety, and the implementation of supportive care for patients with BC is necessary [6, 106]. Historically, pandemics have impacted populations unequally, and this social inequality can exploit populations with chronic disease, including BC [8]. There were evident racial disparities to BC services during the pandemic, which is consistent with research conducted prior to the pandemic [112]. Additional SDH included older age, low SES, health insurance, and area of residence, but due to limited research, more research is needed in this area.

### Implications for policy, practice, and research

Since early detection and timely treatment are essential for optimal prognosis and QoL among BC patients [113], research on the long-term clinical impact of the pandemic on BC diagnoses and prognoses is needed. Understanding the influence of COVID-19 on QoL potentially helps enable the development of patient-centred care, tailored intervention strategies, and support services to reduce long-term physical and psychological morbidity [114]. Additionally, the use telemedicine for non-essential needs can optimize BC care post-pandemic [115] and improve psychosocial well-being [88].

The consequences of the pandemic may impact morbidity and mortality for BC globally; therefore, it is important to use this information to implement timely changes to health-care systems to minimize long-term impacts of the pandemic and improve evidence-based multidisciplinary needs, especially for socially disadvantaged groups. Further studies should be conducted across a broader range of countries, and more comparative population-based research on specific sub-themes, such as screening services or clinical outcomes, is required.

### Strengths and limitations

The included studies provide a comprehensive scope of the early impact of COVID-19 on various countries. However, it is important to note that most research articles included in this study took place in developed countries and the findings may not be transferable to all countries and several studies had relatively low sample sizes. The majority of the studies included in the review were cross-sectional or retrospective; more prospective studies are needed to understand the long-term impact of the COVID-19 pandemic. Other limitations to this review process include the possibility that some relevant articles may have been missed. Many of the identified and eligible journal articles were only published as an abstract, therefore they were not included. As more information on the topic of COVID-19, BC, and SDH becomes available, the scoping review will be updated.

## Conclusion

This scoping review provides a thorough assessment of the impact of the pandemic on BC services and subsequent health outcomes, including QoL, emphasizing the detrimental impact of the pandemic on women with a diagnosis of BC, and for women with pending BC diagnoses. The varying impact to BC care demonstrates how health inequities have been exacerbated during the pandemic by identifying known SDH; therefore, this review has provided evidence of the ‘syndemic’ relationship between COVID-19, BC, and the SDH.

## Data Availability

The data underlying this article are available in the article.

## Appendix 1. Protocol for scoping review: understanding the impact of COVID-19 on breast cancer

### (i) Identifying the research questions

**Table:**

	Embase	15 November 2021
1	“coronavirus”/exp OR coronavirus:ti, ab, de, kw OR “coronavirus infection”/de OR “coronavirinae”/exp OR “coronavirinae” OR “coronaviridae infection”/exp OR “coronaviridae infection” OR “coronavirus disease 2019”/exp OR “Covid 19”:ti, ab, kw “SARS-CoV-2” OR “SARS-CoV-19”	234 733
2	“breast cancer”/exp OR breast NEXT/2 cancer* OR breast NEXT/2 neoplasm*	626 475
3	1 AND 2	1727
4	Limit 3 to HUMANS	1690

**Table:**

	PubMed	15 November 2021
1	“coronavirus”[MeSH] OR “coronavirus infections”[MeSH Terms] OR “coronavirus”[All Fields] OR “covid 2019”[All Fields] OR “SARS2”[All Fields] OR “SARS-CoV-2”[All Fields] OR “SARS-CoV-19”[All Fields] OR “severe acute respiratory syndrome coronavirus 2” [supplementary concept] OR “coronavirus infection”[All Fields] OR “severe acute respiratory pneumonia outbreak”[All Fields] OR “novel cov”[All Fields] OR “2019ncov”[All Fields] OR “sars cov2”[All Fields] OR “cov22”[All Fields] OR “ncov”[All Fields] OR “covid-19”[All Fields] OR “covid19”[All Fields] OR “coronaviridae”[All Fields] OR “corona virus”[All Fields]	215 467
2	“breast neoplasms”[MeSH Terms] OR “breast neoplasm*”[Text Word] OR ‘breast cancer*”[Text Word]	405 400
3	1 AND 2	667
	CINAHL	15 November 2021
1	‘coronavirus disease 2019’ OR ‘covid 19’ OR coronavirus OR “coronavirus infection” OR SARS-CoV-2 OR SARS-CoV-19	42 951
2	‘breast cancer’ OR breast N2 cancer* OR breast N2 neoplasm*	111 617
4	1 AND 2	107
	Cochrane Library	
1	‘coronavirus disease 2019’ OR ‘covid 19’ OR coronavirus OR “coronavirus infection” OR SARS-CoV-2 OR SARS-CoV-19	8484
2	breast NEAR/2 neoplasm* OR Breast NEAR/2 cancer*	39 362
3	1 AND 2	60
	Web of Science	15 November 2021
1	TS = (‘coronavirus disease 2019’ OR ‘covid 19’ OR coronavirus OR “coronavirus infection” OR SARS-CoV-2 OR SARS-CoV-19)	223 900
2	TS = ((breast NEAR/2 neoplasm*) OR (Breast NEAR/2 cancer*))	557 054
3	1 AND 2, limited to articles	566
	APA PsycINFOR	15 November 2021
1	‘coronavirus disease 2019’ OR ‘covid 19’ OR coronavirus OR “coronavirus infection” OR SARS-CoV-2 OR SARS-CoV-19	8221
2	‘breast cancer’ OR breast N2 cancer* OR breast N2 neoplasm*	15 797
4	1 AND 2	13

This aim of this review is to better understand the relationship between COVID-19 and breast cancer; specifically, the impact the pandemic has had on BC services and subsequent health outcomes.

### (ii) Identifying relevant studies

Database searches using Embase, PsycINFO, PubMed, CINAHL, Cochrane Library, and Web of Science included only peer-reviewed articles after 2020. The database search used the following keywords: (pandemic or COVID-19 or coronavirus) and (breast cancer). Here outlines the search strategy conducted by a medical librarian with identified records:

### (iii) Screening identified records

**Excludes:** single case reports, letters to/from editor, editorials, brief commentary, recommendations, non-English, impact on research/clinical trials, non-BC-related, conference, before 2020, manuscripts, specific only to breast reconstruction, diagnosis/prognosis of COVID-19, protocols, genetic testing, guidelines/management, modelling research, pre-prints, male BC, COVID-19 diagnoses, abstracts/posters (saved abstracts in different folder for later full-text inclusion)

**Includes:** specific to BC (including papers with multiple cancers but with distinct BC results), observational studies, qualitative studies, and mixed-methods studies

### (iv) Ongoing screening of relevant studies

To add relevant records with alerts from each database.
